# Primary graft dysfunction in heart transplantation: the challenge to survival

**DOI:** 10.1186/s13019-024-02816-6

**Published:** 2024-06-01

**Authors:** Hüseyin Sicim, Wing Sum Vincy Tam, Paul C. Tang

**Affiliations:** 1 Department of Cardiovascular Surgery, Mayo Clinic, Rochester, MN USA; 2 School of Clinical Medicine, Li Ka Shing Faculty of Medicine, University of Hong Kong, Hong Kong, China

**Keywords:** Primary graft dysfunction, Heart transplantation, Management, Allogreft

## Abstract

Primary graft dysfunction (PGD) is a life-threatening clinical condition with a high mortality rate, presenting as left, right, or biventricular dysfunction within the initial 24 h following heart transplantation, in the absence of a discernible secondary cause. Given its intricate nature, definitive definition and diagnosis of PGD continues to pose a challenge. The pathophysiology of PGD encompasses numerous underlying mechanisms, some of which remain to be elucidated, including factors like myocardial damage, the release of proinflammatory mediators, and the occurrence of ischemia-reperfusion injury. The dynamic characteristics of both donors and recipients, coupled with the inclination towards marginal lists containing more risk factors, together contribute to the increased incidence of PGD. The augmentation of therapeutic strategies involving mechanical circulatory support accelerates myocardial recovery, thereby significantly contributing to survival. Nonetheless, a universally accepted treatment algorithm for the swift management of this clinical condition, which necessitates immediate intervention upon diagnosis, remains absent. This paper aims to review the existing literature and shed light on how diagnosis, pathophysiology, risk factors, treatment, and perioperative management affect the outcome of PGD.

## Introduction

Nowadays, heart transplantation takes its place as the most effective treatment for end-stage heart failure. Globally, an annual average of 5000 heart transplantations are performed [1]. Improvements in current treatment modalities and immunosuppression therapies have significantly improved postoperative survival. However, despite these advancements, transplant recipients remain vulnerable to early postoperative mortality attributed to early allograft failure. In certain individuals, these issues may arise from factors such as hyperacute rejection, pulmonary hypertension, surgical complications, and sepsis. Nevertheless, in the majority of cases, the primary culprit is graft failure resulting from extensive myocardial damage, a condition termed primary graft dysfunction (PGD). However, the lack of clarity in the diagnosis of PGD makes it very difficult to determine the true incidence of this condition.

In the most recent report from the International Society for Heart and Lung Transplantation (ISHLT) registry prior to 2014, the one-month and three-month mortality rates were 10% and 14%, respectively, for a study involving more than 100,000 transplant recipients [2, 3]. Approximately 70% of deaths within the first month postoperatively were attributed to either graft failure or multiple organ failure [2, 3]. Although the exact diagnosis remains unclear, the majority of these early fatalities are likely to be due to PGD. Moreover, significant disparities in the reported incidence and outcomes of PGD were evident across diverse centers (4–5). This wide spectrum of variations may be attributed to several factors, including the utilization of distinct diagnostic criteria for PGD among centers, divergent clinical strategies, as well as variations in donor and recipient acceptance criteria. In response to the pressing need, a workshop convened by the ISHLT resulted in the establishment of a consensus regarding PGD, along with the implementation of a rating system. This consensus represents a milestone in the quest to ascertain the true prevalence of PGD and its impact on postoperative morbidity and mortality. This review aims to provide up-to-date insights and information on the topic, focusing on current recommendations and strategies related to PGD. It underscores the most recent advancements in the definition, pathophysiology, risk factors, and management algorithms of this critical condition.

### Definition and grading of PGD

PGD is defined as graft dysfunction caused by severe ventricular dysfunction within the first 24 h following donor graft transplantation. This condition typically arises without influence from secondary causes such as hyperacute rejection, pulmonary hypertension, or surgical complications [3, 6, 7]. Clinically, it presents as reduced cardiac output, hypotension, and either single or biventricular failure despite adequate filling pressures in the graft [3, 8].

The 2014 ISHLT Consensus workshop classified graft dysfunction into primary and secondary causes, providing clear definition and grading criteria for PGD [3]. This consensus on a standardized definition facilitated future studies to determine the true incidence and investigate the potential multifactorial etiologies contributing to PGD. PGD and secondary graft dysfunction have been defined as completely separate entities. Secondary graft dysfunction necessitates the presence of an underlying cause, such as hyperacute rejection, pulmonary hypertension, or surgical complications.

PGD is divided into two major groups: PGD-LV for isolated left ventricular or biventricular failure, and PGD-RV for isolated right ventricular involvement [3]. PGD-LV is further graded into mild, moderate and severe (Table 1). Mild to moderate PGD-LV criteria include: an LVEF ≤ 40% by echocardiography or hemodynamic indicators for high filling pressures such as RAP > 15 mm Hg, PCWP > 20 mm Hg, and CI < 2.0 L/min/m². Moderate PGD-LV is further diagnosed when additional criteria are met, involving either hypotension with MAP < 70 mm Hg or the need for high-dose inotropes or IABP. The need for left or biventricular mechanical support including ECMO, LVAD or biVAD is diagnostic for severe PGD-LV [3]. PGD-RV is diagnosed by the need for a RVAD or the presence of isolated right ventricular dysfunction, indicated by RAP > 15 mmHg, PCWP < 15 mmHg, CI < 2.0 L/min/m^2^, TPG < 15 mmHg, and /or sPAP < 50 mmHg [3]. PGD-RV does not include further grading.

**Table 1:** Criteria and grading of primary graft dysfunction

		**One of the following criteria:**
	**Mild**	• LVEF ≤ 40% by echocardiography, or • Hemodynamics with RAP > 15 mm Hg, PCWP > 20 mm Hg, • CI < 2.0 L/min/m2 (lasting more than 1 h) requiring low-dose inotropes
		**One criterion from 1 and one criterion from 2**:
PGD-LV	**Moderate**	**1. Criteria** • LVEF ≤ 40%, or • Hemodynamic compromise with • RAP > 15 mm Hg, PCWP > 20 mm Hg, CI < 2.0 L/min/m2 • Hypotension with MAP < 70 mm Hg (> 1 h)
		**2. Criteria** • High-dose inotropes: Inotrope score > 10 or • Newly placed IABP (Regardless of inotropes)
	**Severe**	**Dependence on left or biventricular mechanical support including;**
		• ECMO, LVAD, BiVAD, or percutaneous LVAD
	**Diagnosis requires either both 1 and 2, or 3 alone**:	
PGD-RV	1. Hemodynamics with RAP > 15 mmHg, PCWP < 15 mmHg, CI < 2.0 L/min/m2	
	2. TPG < 15 mmHg and/or sPAP < 50 mm Hg, or	
	3. Need for RVAD	

**BiVAD**, biventricular assist device; **CI**, cardiac index; **ECMO**, extracorporeal membrane oxygenation; **IABP**, intraaortic balloon pump; **LVAD**, left ventricular assist device; **PCWP**, pulmonary capillary wedge pressure; **PGD-LV**, primary graft dysfunction of the left ventricle; **PGD-RV**, primary graft dysfunction of the right ventricle; **RAP**, right atrial pressure; **RVAD**, right ventricular assist device; **sPAP**, pulmonary artery systolic pressure; **TPG**, transpulmonary pressure gradient

### Incidence

In pre-standardization studies, notable discrepancies in incidence reporting, ranging from around 2.5–30%, were observed, primarily attributable to variations in diagnostic criteria and management algorithms [9, 10]. For instance, in a retrospective analysis by Russo et al. utilizing the United Organ Sharing Network (UNOS) database, PGD incidence was reported at approximately 2.5% [7]. However, it is important to note that this study defined PGD as a critical outcome encompassing postoperative mortality or re-transplantation within the initial three months post-transplant. Consequently, it is likely that only the most severe cases were included in the analysis, resulting in a lower reported incidence.

Subsequent studies conducted following the establishment of a standardized definition endorsed as a universal guideline by ISHLT are anticipated to reveal more accurate data. For instance, Sabatino et al. (2017) reported an incidence of PGD in 13% (72 out of 518) of heart transplant recipients, as assessed by ISHLT criteria. Among these, 49% fell into the severe PGD category, with 78% of those patients belonging to the biventricular PGD-LV subgroup, demonstrating a worse prognosis compared to isolated ventricular dysfunction. Regarding mortality risks, mild PGD incurred a 0.5% mortality rate, moderate PGD a 12% rate, and severe PGD a 54% rate [11]. Similarly, Foroutan et al. [12] found an incidence of PGD-LV at 17% (comprising 3.6% mild, 9.5% moderate, and 3.9% severe) in 412 heart transplant recipients, in accordance with ISHLT guidelines. Notably, severe PGD in this cohort carried a one-month mortality rate of 52%. In a separate study, Nicoara et al. reported a PGD incidence of 31% in 312 transplant patients, with significantly higher 30-day mortality rates among patients with PGD [13]. Collectively, these findings underscore that mild, moderate, or severe PGD develops in approximately 2.5–30% of heart transplant recipients, with severe PGD associated with a poor prognosis. Moayedi et al. showed that the incidence of PGD seems to be increasing, but that this was especially due to changes in non-severe PGD. However more liberal and widespread use of postoperative ECMO inevitably leads to an increase in the incidence severe PGD due to the definition of PGD [14].

Although PGD-LV is a widely discussed topic, the incidence of PGD-RV may be unaware Kaveevorayan et al. reviewed a total of 122 consecutive patients who underwent HTX in 2023. Primary isolated RV failure (PI-RVF) was present in 65 of 111 patients (59%) and 31 (48%) met the criteria for PGD-RV. Among patients with mild and moderate PI-RVF, patients who did not meet the criteria of PGD-RV. Therefore, they advised a revised definition of PGD-RV may be needed since patients who had adverse outcomes did not meet the criteria of PGD-RV. Moreover, as postoperative ECMO is the major defining criterium for severe PGD-LV, the treatment of RV failure with ECMO may lead to an underestimation of the incidence of PGD-RV [15].

It is noteworthy that some centers may adopt a more aggressive approach to postoperative patient management, including a tendency to use mechanical circulatory support. Implementing support interventions in cases lacking indications or employing an overly aggressive approach could potentially result in an overestimation of post-transplant PGD incidence.

### Pathophysiology of PGD

In the perioperative phase, the donor heart is susceptible to various forms of damages resulting from brain death, cold ischemia during transport, warm ischemia during implant surgery, and reperfusion. Additionally, systemic factors within the recipient’s body pose additional risks to the donor heart’s function.

### Brain death

The etiology of brain death is usually associated with intercerebral hemorrhage, hypoxia, or an increase in intracranial pressure secondary to inflammation. These events can lead to brain herniation and subsequent pons ischemia due to brain edema within the confined cranial space [16]. Consequently, immediate and substantial release of norepinephrine from the adrenal medulla occurs following brain death. This norepinephrine surge stimulates baroreceptors, resulting in the Cushing’s triad, clinically marked by hypertension, bradycardia, and irregular respiratory patterns [17, 18]. Furthermore, this norepinephrine release induces mitochondrial and cytosolic calcium accumulation at the cellular level [19]. Loss of sympathetic activity in the spinal cord results in profound vasodilation, leading to reduced preload and afterload, and therefore compromised myocardial perfusion. Early administration of vasopressors is pivotal in mitigating unopposed vasodilation [20]. . Additionally, hormonal alterations, such as decreased serum levels of thyroid hormones, cortisol, and insulin, lead to reduced myocardial contractility [21].

### Hypothermic ischemia

During the removal of the donor heart, cardiac arrest is induced by infusing a cold cardioplegia solution at approximately 4 °C while the aorta is cross-clamped. Cold storage remains the primary method for transporting donor hearts. Following procurement, the donor heart is placed in cold preservation solutions, surrounded by ice to maintain hypothermia. Hypothermia significantly slows down cellular metabolism but does not completely stop it. Hypothermia within the range of 0–4 °C reduces the metabolic rate by approximately 12-fold [21]. Prolonged static storage inevitably results in ischemic damage due to the continued albeit slowed metabolic processes. The intracellular acidic environment activates the Na+/H + pump, facilitating the removal of excess H + ions from the cell [22]. Elevated intracellular sodium (Na+) levels disrupt intracellular calcium (Ca2+) accumulation through the Na+/Ca2 + pump, a pathway pivotal in cellular damage. Older donors are more susceptible to ischemic injury [23], likely attributed to their medical history, including hypertension, coronary artery disease, or left ventricular hypertrophy [24].

It is also important to use new technological devices to make the hypothermia process most beneficial. The impact of donor organ preservation conditions on severity of PGD and survival has not been well characterized. The 2018 FDA clearance of a novel system for controlled, hypothermic organ preservation, the Paragonix SherpaPak Cardiac Transport System (Paragonix Technologies, Inc., Waltham, MA). In 2024, D’Alessandro et al. showed that the controlled hypothermic preservation was associated with a significant reduction in the incidence of severe PGD compared to ice (6.6% [37/559] vs. 10.4% [47/452], *p* = 0.039) [25]. Positive results obtained from new technologies may be promising for the future.

### Warm ischemia (Surgical Implant time)

Warm ischemic time is characterized as the duration between the removal of the donor heart from static cold storage and the removal of aortic cross-clamping. During this process, the heart is exposed to higher temperatures, resulting in a gradual rise in metabolic activity and the subsequent generation of free radicals, which cause cellular damage. Banner et al. identified warm ischemic time as an independent risk factor for 30-day mortality [26]. It is an inevitable that the prolongation of this process due to various reasons contributes to an increased likelihood of PGD development.

### Ischemia-reperfusion injury (IRI)

Reperfusion of oxygenated blood to a previously oxygen-deprived heart results in calcium overload and the generation of free oxygen radicals, which impair the function of cellular enzymes [27]. The substantial calcium load, particularly in energy-demanding cardiac tissue, activates the formation of non-specific mitochondrial permeability transition pores (mPTP) in the mitochondrial membrane. mPTP facilitates specific cell death pathways; for instance, cytochrome C entry induces apoptosis, while water influx leads to cellular swelling and necrosis. Mechanisms that impede mPTP formation may play an important role in mitigating IRI. While some publications have suggested that cyclosporine, a known mPTP desensitizer, may offer protection against ischemic damage from myocardial infarction, a meta-analysis by Upadhaya et al. found that cyclosporine did not impact postoperative morbidity and mortality [28]. However, in an animal study on mPTP, Zhang et al. achieved promising and selective outcomes using mitochondria-targeted nanoparticles as a treatment approach [29]. The ongoing advancement of nanotechnology holds promise for future cellular-level therapeutic strategies.

### Recipient factors

Recipient factors may also contribute to the development of PGD. The likely mechanism involves the recipient experiencing an activation of the systemic inflammatory response, resulting in a vasodilated systemic circulation unresponsive to conventional vasopressor support [30]. Reasons for this vasoplegic response include pre-transplant mechanical circulatory support, prolonged cross-clamp time, and large transfusion requirements [31]. Although the precise role of this response in PGD pathophysiology remains incompletely understood, it is plausible that the mechanism involves the overproduction of nitric oxide or other endogenous vasodilators and the release of multiple proinflammatory cytokines, ultimately leading to the upregulation of nitric oxide synthase [30, 32].

### Risk factors for PGD

Multiple risk factors associated with the development of PGD have been consistently identified in published literature over time. Broadly, these factors can be categorized into donor, procedural, and recipient factors, as summarized in Table 2. The high demand of heart transplantation, coupled with the use of marginal and expanded donor criteria, has placed growing pressure on heart transplant programs to use available donor hearts. Additionally, the increasing success of heart transplantation has led to more expanded donor criteria, encompassing older patients and those with more comorbidities. The increasing prevalence of risk factors for PGD, particularly donor and recipient age, is likely a significant contributor to the high incidence of PGD as reported in recent ISHLT registry data.

**Table 2:** Risk factors for PGD

Donor Factors	Recipient Factors	Procedural Factors
• Older Age	• Older Age	• Long ischemic time
>20 years	• High weight	• Long CPB time
• Gender mismatch	• Mechanical support	• Weight mismatch
Female donor/male recipient	VAD/ECMO	Low weight donor heart
• Cause of death	• Congenital heart disease	• Heart team experience
- Intracranial hemorrhage	• Re-sternotomies	• Center volume
• High inotropic requirements	• Comorbidities	• Massive blood transfusion
-Noradrenaline	-Renal/Liver dysfunction	• Emergency transplant
• Cardiac dysfunction	• Ventilator dependence	
• Comorbidities (DM, HT)	• Multiorgan transplant	
• Left ventricular hypertrophy	• Elevated PVR	
• Drug abuse	• Infection	
• Infection	• Retransplant • Amiodarone use	

**CPB**, cardiopulmonary bypass; **DM**, diabetes mellitus; **ECMO**, extracorporeal membrane oxygenation; **HT**, hypertension; **PVR**, pulmonary vascular resistance; **VAD**, ventricular assist device

In a large UNOS registry analysis, Russo et al. investigated the association between donor age and ischemic time in terms of post-transplant mortality [5]. The findings revealed that hearts from donors under 20 years of age can endure ischemic time exceeding six hours, whereas the survival is reduced when hearts from donors over 33 years of age exceed 3.5 h of ischemic time. Avtaar Singh et al., in their own study, emphasized the significance of donor age as a pivotal risk factor in a multivariate analysis [6]. Notably, older donor hearts subjected to prolonged ischemia exhibit a higher degree of ischemic damage compared to their younger counterparts. Their study concluded that for every ten-year increase in donor heart age, the risk is increased by 20%.

Due to donor shortages, prolonged waiting times are inevitable for recipients. During this waiting period, preoperative mechanical circulatory support may be deemed necessary as a bridge to transplantation for patients with a poor prognosis. However, research indicates that the use of preoperative ECMO or VAD significantly elevates the risk of postoperative PGD [5, 9]. A recent single-center study conducted by Nicoara et al. reported a trend towards increased PGD in preoperative LVAD recipients (40% versus 32%, *P* = 0.05) [15]. Technological advancements, particularly in continuous-flow LVADs (CF-LVADs), have substantially contributed to the widespread success of advanced heart failure treatment, with approximately 95% of VAD implants now being CF-LVADs. Truby et al., in a recent study, emphasized the significant association between continuous-flow LVAD (CF-LVAD) use and the risk of severe PGD. Notably, 80% of the 56 patients with severe PGD in their study had undergone preoperative CF-LVAD implantation [33].

Furthermore, the preoperative administration of amiodarone is associated with an increased incidence of severe PGD. In a recent single-center study of 269 heart transplant cases, pre-transplant use of amiodarone was associated with a higher incidence of severe PGD within the first 24 h post-transplant (20% versus 5%, *P* < 0.001). This incidence of severe PGD was directly proportional to the dosage administered [33]. Reducing the dose of amiodarone may reduce the risk of severe PGD; however, this decision should be made taking into account the patient’s clinical need. This finding was corroborated by an analysis of the ISHLT, which reported an increased one-year mortality rate associated with pre-transplant amiodarone use [34].

Efforts to assess risk factors across various categories often yield results influenced by diverse criteria employed by different medical centers. Therefore, there has always been a need for a consistent risk scoring system. Currently, the only valid scoring system for predicting PGD is the RADIAL score [32]. This scoring model was derived from a multivariate analysis of independent risk factors for PGD in a single-center cohort study of 621 heart transplants. The RADIAL score encompasses six distinct factors, four of which pertain to the recipient and two to the donor. The recipient-related factors include right atrial pressure exceeding 10 mm Hg, age over 60 years, diabetes, and dependence on inotropic support. Donor-related factors encompass age over 30 years and ischemia duration exceeding 240 min. Each presence of these factors in an individual patient contributes one point to the final score.

In their 2013 follow-up study, the same authors examined the incidence of PGD in a cohort of 698 heart transplantations performed in Spain, reaffirming the validity of the RADIAL score [35]. This investigation revealed an overall incidence of moderate to severe PGD cases at 22%. Among these PGD cases, 55% exhibited left ventricular dysfunction, typically as part of biventricular failure. Half of the diagnosed patients received treatment with a mechanical assist device, resulting in a 30-day mortality rate of 40%. The study also highlighted that while PGD is diagnosed within the initial 30 days, the mortality process may extend over several months due to factors such as multiorgan failure and sepsis. The RADIAL model classifies PGD into three risk groups: low (0–1 points), medium (2 points), and high (3 points or above), with respective incidence of 12%, 19%, and 28%. It is worth noting that during the development of this scoring system, ventricular assist devices (VADs) were not as comman as they are today. Therefore, increasing VAD treatments in recent years may lead to a need for changes in current scoring systems.

In the international consortium on primary graft dysfunction published in 2023, which was prepared by ten centers in the United States, Canada and Europe and included a total of 2746 patients, they evaluated the performance of the most strongly validated PGD risk tool, the RADIAL score, in a contemporary cohort;. 215 participants (7.8%) met the criteria for severe PGD. There was an increase in the incidence of severe PGD over the study period (P value for trend by difference sign test = 0.004). The RADIAL score performed poorly in our contemporary cohort and was not associated with severe PGD; it had an AUC of 0.53 (95% CI 0.48–0.58) [14]. Therefore, the reliability of the RADIAL score is still a matter of debate.

### Treatment and management of PGD

PGD treatment and management poses significant challenge. Nonetheless, the extensive adoption of mechanical circulatory support (MCS), alongside positive inotropes and pulmonary vasodilators, represents a pivotal advancement in heart transplantation surgery. MCS holds a crucial role not only preoperatively but also postoperatively. It serves to avert multiorgan failure in critically ill patients and facilitate the recovery of cardiac allograft during the rehabilitation phase.

The increasing utilization of venoarterial extracorporeal membrane oxygenation (VA-ECMO) is notable due to its efficacy and ease of application when compared to ventricular assist devices (VADs). In a study conducted by Takeda et al., superior outcomes were observed with VA-ECMO in patients experiencing PGD following heart transplantation. Within a cohort of 597 heart transplant recipients, PGD occurred in 7.4% (44 patients). Among these PGD cases, 17 received VAD support, while 27 were supported by VA-ECMO. Although there was no significant difference in terms of mortality between the two groups, the VA-ECMO cohort exhibited advantages in terms of reduced major bleeding events, lower incidence of renal failure, and a reduced need for prolonged inotropic support [36]. In a separate study by D’Alessandro et al., among 90 patients who developed graft dysfunction within 48 h post-transplant, the survival rate was 48% in the ECMO group, in contrast to 25% in the VAD group [9].

Regarding cannulation strategies in ECMO for postcardiotomy shock, there is no discernible distinction between standard approaches. Both central and peripheral cannulation techniques can be employed as viable configuration options. Several studies have reported no significant disparity in outcomes between these two cannulation methods [37]. Based on our clinical experience, peripheral cannulation is preferrable due to its minimally invasive nature for patients. Peripheral cannulation is generally considered safewhen performed using surgical vessel exploration through the femoral artery and vein. Olivella et al. reported that early initiation of ECMO did not benefit the HTx recipient compared to a more delayed ECMO initiation. However, they did notice a survival benefit in peripheral ECMO compared to central ECMO [38]. Moreover, another recent study suggested some slight benefits in the use of axillary instead of femoral cannulation in post-Tx ECMO [39]. For a comprehensive assessment of peripheral cannulation configurations and strategies, the expert consensus guide jointly developed by EACTS/STS/AATS can serve as a valuable resource [40].

Recent studies consistently support the use of MCS in severe PGD for management. Jacob et al. conducted a study in 2019 involving 1030 patients, revealing that 3% of them developed severe PGD requiring MCS. Remarkably, 81% of these patients treated with VA-ECMO achieved successful weaning [41].

**Fig. 1:**
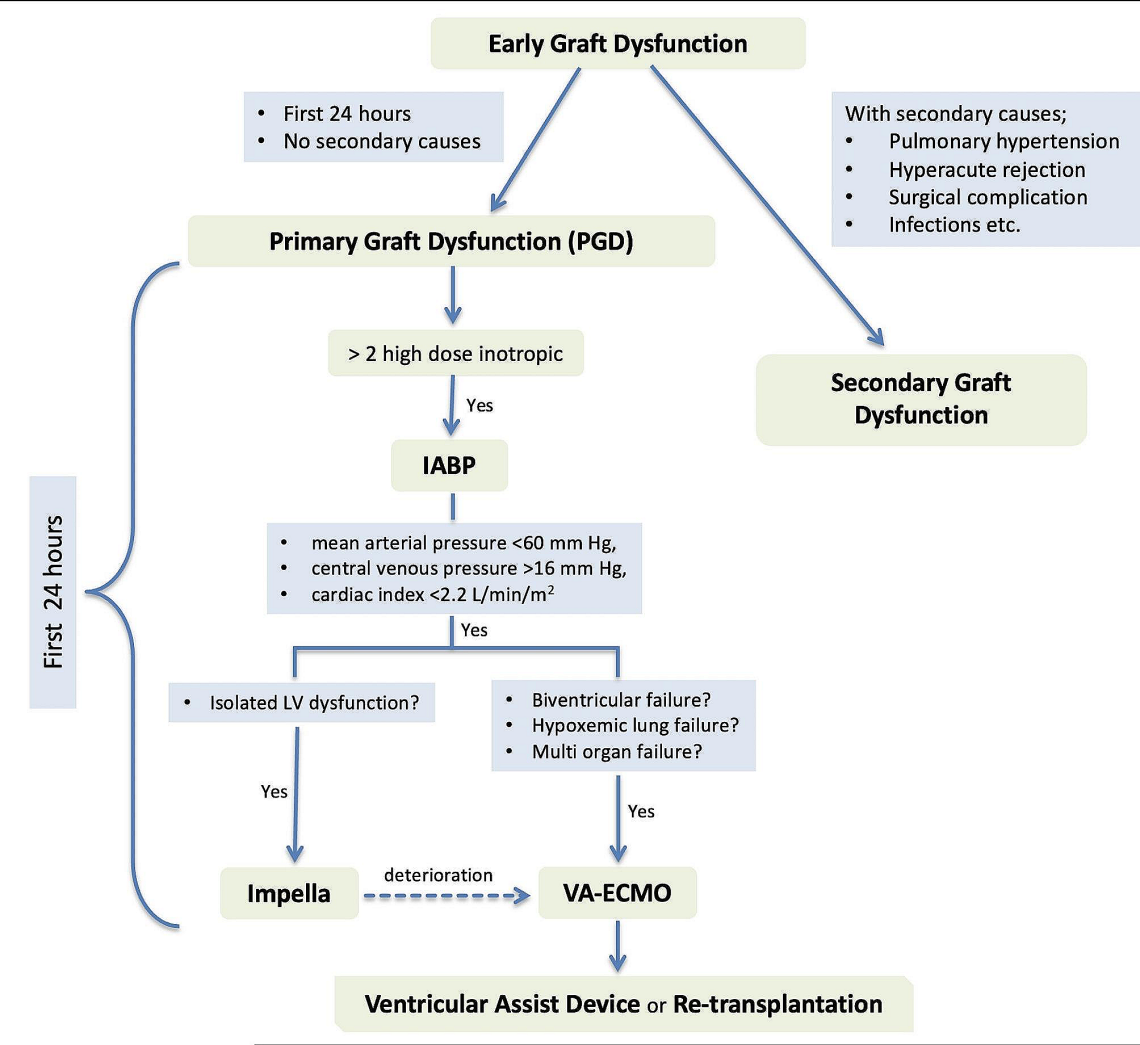
Management of PGD

The critical question revolves around the optimal timing for diagnosing and treating severe PGD post-heart transplant. In a 2019 study by DeRoo et al., PGD was identified in 38 (10.5%) of 362 heart transplant recipients [42]. These patients were stratified into two groups: conservative ECMO comprising 18 patients and prompt ECMO involving 20 patients. Under the prompt ECMO protocol, implemented after 2015, ECMO was initiated in the operating room if patients maintained acceptable hemodynamics (mean arterial pressure > 60 mm Hg, central venous pressure < 16 mm Hg, and cardiac index > 2.2 L/min/m²) despite the use of more than 2 high-dose inotropes post-cardiopulmonary bypass. Conversely, the conservative ECMO approach, used before 2015, applied ECMO in the intensive care unit for patients unable to regain adequate hemodynamics despite high-dose inotropes. Although no differences were observed in terms of ICU stay or major complications, in-hospital mortality decreased significantly from 28% (conservative) to 5% (prompt). This study underscores the benefits of early VA-ECMO in promoting myocardial recovery and reducing mortality in patients with severe PGD without increasing the risk of complications [43]. In accordance with established clinical principles and experience, the pivotal step in post-transplant patient management is deemed to be the application of ECMO at the right time with the right indications. The algorithm we have developed for graft dysfunction is presented in Fig. 1 for reference.

In recent years, the increasing demand for donors and improved transplant success rates have led to a greater utilization of marginal and high-risk donor hearts by surgeons. It is imperative to conduct studies that shed light on the incidence of PGD in recipients of marginal donor cardiac allografts and offer effective post-transplant management strategies. Lima et al. analyzed 260 heart transplant cases, with 53 involving the use of marginal donor cardiac allografts. The recipients from the alternative list were notably older and had a higher prevalence of comorbidities such as diabetes mellitus and ischemic cardiomyopathy. While overall mortality rates were higher in the alternative list, intriguingly, there was no significant difference in PGD incidence between the two groups [43]. Moreover, despite the early negative consequences of PGD, its long-term effect does not seem to be very negative. Settepani et al. confirmed that PGD is associated with poor in-hospital outcome in their study. The poor outcome does not extend beyond the first month of follow-up, with comparable survival between patients with none/mild PGD and moderate/severe PGD in the short and long-term [44].

The increasing utilization of circulatory post-death (DCD) hearts presents a noteworthy concern. This trend is expected to increase the risk of PGD due to prolonged warm ischemic times. Consequently, there is a pressing need for comprehensive research on PGD development in the context of DCD hearts. Presently, data on PGD occurrence among DCD and DBD recipients remain limited. In a comparative study involving 28 patients conducted by Messer et al., no discernible differences were observed in terms of 90-day survival rates. Additionally, there were no statistically significant distinctions between the two groups in variables such as hospital stay duration, rejection rates, allograft function, and 1-year survival rates (DCD, 86%; DBD, 88%; *p* = 0.98) [45]. Ayer et al., compared the incidence, severity and outcomes of patients experiencing PGD after DCD compared to DBD heart transplantation. A total of 459 patients underwent isolated heart transplantation during the study period, the incidence of moderate or severe PGD in DCD and DBD recipients was 34% and 23%, respectively (*P* = 0.070). DCD recipients were more likely to experience severe biventricular PGD than DBD recipients. Among patients with severe PGD, DCD recipients experienced shorter median duration of post-transplant mechanical circulatory support. Their study showed that DCD heart transplant recipients were more likely to experience severe, biventricular PGD than DBD recipients [46]. These findings underscore the comparability of DCD heart donation to conventional DBD heart transplants, potentially expanding the pool of heart transplant candidates in well-selected cases.

## Conclusion

While significant advancements have been made in post-heart transplant follow-up and treatment, early postoperative PGD remains a prominent concern. PGD represents a notable contributor to early mortality following heart transplantation, and its incidence is estimated to affect approximately 20–30% of heart transplant recipients, although precise figures are lacking. Furthermore, the incidence of PGD is on the rise and may continue to increase, particularly due to factors such as increasing age of both donors and recipients, growing utilization of marginal and high-risk donors as well as strategies like DCD transplantation [11].

Effectively managing and preventing PGD necessitates a comprehensive approach that addresses donor, recipient, and procedural risk factors. Consequently, achieving consensus and implementing well-structured algorithms for PGD treatment is imperative. Managing severe PGD presents a formidable challenge, and favorable outcomes are attainable through advanced mechanical circulatory support devices. Early application of VA-ECMO forms a cornerstone of treatment, significantly contributing to early myocardial recovery and enhancing overall survival. However, success in combating PGD hinges on the collective sharing of experiences and the adoption of standardized algorithms encompassing diagnosis, treatment, and perioperative patient management strategies.

## Data Availability

No datasets were generated or analysed during the current study.

## References

[1] Lund LH, Khush KK, Cherikh WS, Goldfarb S, Kucheryavaya AY, Levvey BJ (2017). The Registry of the International Society for Heart and Lung Transplantation: thirty-fourth adult heart transplantation Report-2017; focus theme: allograft ischemic time. J Heart lung Transplantation: Official Publication Int Soc Heart Transplantation.

[2] Lund LH, Edwards LB, Kucheryavaya AY, Dipchand AI, Benden C, Christie JD (2013). The Registry of the International Society for Heart and Lung Transplantation: Thirtieth Official Adult Heart Transplant Report–2013; focus theme: age. J Heart lung Transplantation: Official Publication Int Soc Heart Transplantation.

[3] Kobashigawa J, Zuckermann A, Macdonald P, Leprince P, Esmailian F, Luu M (2014). Report from a consensus conference on primary graft dysfunction after cardiac transplantation. J Heart lung Transplantation: Official Publication Int Soc Heart Transplantation.

[4] D’Ancona G, Santise G, Falletta C, Pirone F, Sciacca S, Turrisi M (2010). Primary graft failure after heart transplantation: the importance of donor pharmacological management. Transpl Proc.

[5] Listijono DR, Watson A, Pye R, Keogh AM, Kotlyar E, Spratt P (2011). Usefulness of extracorporeal membrane oxygenation for early cardiac allograft dysfunction. J Heart lung Transplantation: Official Publication Int Soc Heart Transplantation.

[6] Avtaar Singh SS, Banner NR, Rushton S, Simon AR, Berry C, Al-Attar N (2019). ISHLT primary graft dysfunction incidence, risk factors, and outcome: a UK National Study. Transplantation.

[7] Rhee Y, Kim HJ, Kim JJ, Kim MS, Lee SE, Yun TJ (2021). Primary graft dysfunction after isolated heart transplantation - incidence, risk factors, and clinical implications based on a single-center experience. Circulation Journal: Official J Japanese Circulation Soc.

[8] Iyer A, Kumarasinghe G, Hicks M, Watson A, Gao L, Doyle A (2011). Primary graft failure after heart transplantation. J Transplantation.

[9] D’Alessandro C, Aubert S, Golmard JL, Praschker BL, Luyt CE, Pavie A (2010). Extra-corporeal membrane oxygenation temporary support for early graft failure after cardiac transplantation. Eur J cardio-thoracic Surgery: Official J Eur Association Cardio-thoracic Surg.

[10] Dronavalli VB, Rogers CA, Banner NR (2015). Primary cardiac allograft dysfunction-validation of a clinical definition. Transplantation.

[11] Sabatino M, Vitale G, Manfredini V, Masetti M, Borgese L, Maria Raffa G (2017). Clinical relevance of the International Society for Heart and Lung Transplantation consensus classification of primary graft dysfunction after heart transplantation: Epidemiology, risk factors, and outcomes. J Heart lung Transplantation: Official Publication Int Soc Heart Transplantation.

[12] Foroutan F, Alba AC, Stein M, Krakovsky J, Chien KGW, Chih S (2019). Validation of the International Society for Heart and Lung Transplantation primary graft dysfunction instrument in heart transplantation. J Heart lung Transplantation: Official Publication Int Soc Heart Transplantation.

[13] Nicoara A, Ruffin D, Cooter M, Patel CB, Thompson A, Schroder JN (2018). Primary graft dysfunction after heart transplantation: incidence, trends, and associated risk factors. Am J Transplantation: Official J Am Soc Transplantation Am Soc Transpl Surg.

[14] Moayedi Y, Truby LK, Foroutan F, Han J, Guzman J, Angleitner P, Sabatino M, Felius J, VAN Zyl JS, Rodenas-Alesina E, Fan CP, Devore AD, Miller R, Potena L, Zuckermann A, Farrero M, Chih S, Farr M, Hall S, Ross HJ, Khush KK. The International Consortium on Primary Graft Dysfunction: Redefining Clinical Risk Factors in the Contemporary Era of Heart Transplantation. J Card Fail. 2023 Oct 30:S1071-9164(23)00382-2. 10.1016/j.cardfail.2023.09.018. 10.1016/j.cardfail.2023.09.018 37907150

[15] Kaveevorayan P, Tokavanich N, Kittipibul V, Lertsuttimetta T, Singhatanadgige S, Ongcharit P, Sinphurmsukskul S, Ariyachaipanich A, Siwamogsatham S, Thammanatsakul K, Sritangsirikul S, Puwanant S (2023). Primary isolated right ventricular failure after heart transplantation: prevalence, right ventricular characteristics, and outcomes. Sci Rep.

[16] Smith M (2004). Physiologic changes during brain stem death–lessons for management of the organ donor. J Heart lung Transplantation: Official Publication Int Soc Heart Transplantation.

[17] Dictus C, Vienenkoetter B, Esmaeilzadeh M, Unterberg A, Ahmadi R (2009). Critical care management of potential organ donors: our current standard. Clin Transplant.

[18] Shivalkar B, Van Loon J, Wieland W, Tjandra-Maga TB, Borgers M, Plets C (1993). Variable effects of explosive or gradual increase of intracranial pressure on myocardial structure and function. Circulation.

[19] Souter MJ, Eidbo E, Findlay JY, Lebovitz DJ, Moguilevitch M, Neidlinger NA (2018). Organ Donor Management: part 1. Toward a Consensus to Guide Anesthesia services during Donation after Brain Death. Semin Cardiothorac Vasc Anesth.

[20] Rosendale JD, Kauffman HM, McBride MA, Chabalewski FL, Zaroff JG, Garrity ER (2003). Hormonal resuscitation yields more transplanted hearts, with improved early function. Transplantation.

[21] Belzer FO, Southard JH (1988). Principles of solid-organ preservation by cold storage. Transplantation.

[22] Marasco SF, Kras A, Schulberg E, Vale M, Lee GA. Impact of warm ischemia time on survival after heart transplantation. Transpl Proc. 2012;44(5):1385–9. 10.1016/j.transproceed.2011.12.075 22664020

[23] Marelli D, Laks H, Fazio D, Moore S, Moriguchi J, Kobashigawa J (2000). The use of donor hearts with left ventricular hypertrophy. J Heart lung Transplantation: Official Publication Int Soc Heart Transplantation.

[24] Anaya-Prado R, Delgado-Vázquez JA (2008). Scientific basis of organ preservation. Curr Opin Organ Transplant.

[25] D’Alessandro D, Schroder J, Meyer DM, Vidic A, Shudo Y, Silvestry S, Leacche M, Sciortino CM, Rodrigo ME, Pham SM, Copeland H, Jacobs JP, Kawabori M, Takeda K, Zuckermann A. Impact of controlled hypothermic preservation on outcomes following heart transplantation. J Heart Lung Transpl 2024 Mar 19:S1053–2498(24)01530–4. doi: 10.1016/j.healun.2024.03.010. Epub ahead of print. PMID: 38503386. 10.1016/j.healun.2024.03.010 38503386

[26] Banner NR, Thomas HL, Curnow E, Hussey JC, Rogers CA, Bonser RS (2008). The importance of cold and warm cardiac ischemia for survival after heart transplantation. Transplantation.

[27] Braunwald E, Kloner RA (1985). Myocardial reperfusion: a double-edged sword?. J Clin Investig.

[28] Upadhaya S, Madala S, Baniya R, Subedi SK, Saginala K, Bachuwa G (2017). Impact of cyclosporine a use in the prevention of reperfusion injury in acute myocardial infarction: a meta-analysis. Cardiol J.

[29] Zhang CX, Cheng Y, Liu DZ, Liu M, Cui H, Zhang BL (2019). Mitochondria-targeted cyclosporin A delivery system to treat myocardial ischemia reperfusion injury of rats. J Nanobiotechnol.

[30] Levin RL, Degrange MA, Bruno GF, Del Mazo CD, Taborda DJ, Griotti JJ (2004). Methylene blue reduces mortality and morbidity in vasoplegic patients after cardiac surgery. Ann Thorac Surg.

[31] Patarroyo M, Simbaqueba C, Shrestha K, Starling RC, Smedira N, Tang WH (2012). Pre-operative risk factors and clinical outcomes associated with vasoplegia in recipients of orthotopic heart transplantation in the contemporary era. J Heart lung Transplantation: Official Publication Int Soc Heart Transplantation.

[32] Wang Y, Liu H, McKenzie G, Witting PK, Stasch JP, Hahn M (2010). Kynurenine is an endothelium-derived relaxing factor produced during inflammation. Nat Med.

[33] Wright M, Takeda K, Mauro C, Jennings D, Kurlansky P, Han J (2017). Dose-dependent association between amiodarone and severe primary graft dysfunction in orthotopic heart transplantation. J Heart lung Transplantation: Official Publication Int Soc Heart Transplantation.

[34] Cooper LB, Mentz RJ, Edwards LB, Wilk AR, Rogers JG, Patel CB (2017). Amiodarone use in patients listed for heart transplant is associated with increased 1-year post-transplant mortality. J Heart lung Transplantation: Official Publication Int Soc Heart Transplantation.

[35] Cosío Carmena MD, Gómez Bueno M, Almenar L, Delgado JF, Arizón JM, González Vilchez F (2013). Primary graft failure after heart transplantation: characteristics in a contemporary cohort and performance of the RADIAL risk score. J Heart lung Transplantation: Official Publication Int Soc Heart Transplantation.

[36] Takeda K, Li B, Garan AR, Topkara VK, Han J, Colombo PC (2017). Improved outcomes from extracorporeal membrane oxygenation versus ventricular assist device temporary support of primary graft dysfunction in heart transplant. J Heart lung Transplantation: Official Publication Int Soc Heart Transplantation.

[37] Rastan AJ, Dege A, Mohr M, Doll N, Falk V, Walther T (2010). Early and late outcomes of 517 consecutive adult patients treated with extracorporeal membrane oxygenation for refractory postcardiotomy cardiogenic shock. J Thorac Cardiovasc Surg.

[38] Olivella A, Almenar-Bonet L, González-Vilchez F, Díez-López C, Díaz-Molina B, Blázquez-Bermejo Z, Sobrino-Márquez JM, Gómez-Bueno M, Garrido-Bravo IP, Barge-Caballero E, Farrero-Torres M, García-Cosio MD, Blasco-Peiró T, Pomares-Varó A, Muñiz J, González-Costello J (2023). Mechanical circulatory support in severe primary graft dysfunction: peripheral cannulation but not earlier implantation improves survival in heart transplantation. J Heart Lung Transpl.

[39] Ohira S, Dhand A, Hirani R, Martinez S, Lanier GM, Levine A, Pan S, Aggarwal-Gupta C, Gass AL, Wolfe K, Spielvogel D, Kai M (2023). Cannulation-related adverse events of peripheral veno-arterial extracorporeal membrane oxygenation support in heart transplantation: Axillary versus femoral artery cannulation. Clin Transpl.

[40] Lorusso R, Whitman G, Milojevic M, Raffa G, McMullan DM, Boeken U (2021). 2020 EACTS/ELSO/STS/AATS expert consensus on post-cardiotomy extracorporeal life support in adult patients. Eur J cardio-thoracic Surgery: Official J Eur Association Cardio-thoracic Surg.

[41] Jacob S, Lima B, Gonzalez-Stawinski GV, El-Sayed Ahmed MM, Patel PC, Belli EV (2019). Extracorporeal membrane oxygenation as a salvage therapy for patients with severe primary graft dysfunction after heart transplant. Clin Transplant.

[42] DeRoo SC, Takayama H, Nemeth S, Garan AR, Kurlansky P, Restaino S (2019). Extracorporeal membrane oxygenation for primary graft dysfunction after heart transplant. J Thorac Cardiovasc Surg.

[43] Lima B, Rajagopal K, Petersen RP, Shah AS, Soule B, Felker GM (2006). Marginal cardiac allografts do not have increased primary graft dysfunction in alternate list transplantation. Circulation.

[44] Settepani F, Pedrazzini GL, Olivieri GM, Merlanti B, Cannata A, Lanfranconi M, Frigerio M, Russo CF (2022). Long-term effects of primary graft dysfunction after heart transplantation. J Card Surg.

[45] Messer S, Page A, Axell R, Berman M, Hernandez-Sanchez J, Colah S (2017). Outcome after heart transplantation from donation after circulatory-determined death donors. J Heart lung Transplantation: Official Publication Int Soc Heart Transplantation.

[46] Ayer A, Truby LK, Schroder JN, Casalinova S, Green CL, Bishawi MA, Bryner BS, Milano CA, Patel CB, Devore AD (2023). Improved outcomes in severe primary graft dysfunction after Heart Transplantation following Donation after Circulatory Death compared with Donation after Brain Death. J Card Fail.

